# Noninvasive prenatal testing in routine clinical practice for a high-risk population: Experience from a center

**DOI:** 10.1097/MD.0000000000005126

**Published:** 2016-10-14

**Authors:** Guijie Qi, Jianping Yi, Baosheng Han, Heng Liu, Wanru Guo, Chong Shi, Lirong Yin

**Affiliations:** a Department of Obstetrics and Gynecology, The Second Hospital of Tianjin Medical University, Tianjin; b Department of Genetics; c Department of Obstetrics and Gynecology, Tangshan Maternal and Children Health Hospital, Tangshan City, Hebei, China.

**Keywords:** chromosomal abnormalities, maternal serum screening, noninvasive prenatal testing, ultrasound screening

## Abstract

This study aimed to summarize the effects of noninvasive prenatal testing (NIPT) on aneuploidy among high-risk participants in Tangshan Maternal and Children Health Hospital.

NIPT or invasive prenatal diagnosis was recommended to patients with a high risk of fetal aneuploidy from February 2013 to February 2014. Patients who exhibited eligibility and applied for NIPT from January 2012 to January 2013 were included in a comparison group. The rates of patients who underwent invasive testing, declined to undergo further testing, and manifested trisomies 21, 18, and 13 were compared between two groups. Follow-up data were obtained from the participants who underwent NIPT from 2013 to 2014.

A total of 7223 patients (3018 and 4205 individuals before and after NIPT) were eligible for analysis. After NIPT was introduced in 2013 to 2014, 727 patients (17.3%) underwent invasive testing, 2828 preferred NIPT (67.3%), and 650 declined to undergo further testing (15.5%). A total of 34 cases of trisomies 21, 18, and 13 (0.8%) were found. In 2012 to 2013, 565 patients (18.7%) underwent invasive testing and 2453 declined to undergo further testing (81.3%). A total of 7 cases of trisomies 21, 18, and 13 were documented (0.2%). Of these cases, 24 were found from NIPT and 10 cases were found from invasive testing. The number of participants who declined to undergo further testing significantly decreased after NIPT was introduced (81.3% vs. 15.5%, *P* < 0.001). The sensitivity and specificity of NIPT for trisomies 21, 18, and 13 were 100% and 99.9%, respectively. The detection rates of NIPT for trisomies 21, 18, and 13 also significantly increased (0.2% vs. 0.8%, *P* < 0.001). By contrast, the overall rates of invasive testing remained unchanged (18.7% vs. 17.3%, *P* = 0.12). The positive predictive values of NIPT for trisomies 21, 18, and 13 were 100%, 83.3%, and 50.0%, respectively. The false positive rates of NIPT were 0% and 0.04%.

With NIPT implementation in clinical practice, the rate of declining a follow-up test among high-risk women was decreased and the detection rate of prenatal chromosomal aneuploidy for trisomies 21, 18, and 13 was increased without requiring numerous invasive procedures.

## 1 Introduction

Noninvasive prenatal testing (NIPT) is a novel technology used to detect trisomies 21, 18, and 13 by analyzing cell-free DNA in maternal circulation.^[1]^ NIPT exhibits high sensitivity and specificity and provides negative predictive values for autosomal trisomies.^[2–4]^ However, independent data on the performance of NIPT in actual clinical practice are limited,^[5,6]^ and women's preferences for daily clinical care are rarely investigated.^[2,7]^ Two independent studies have shown that the probability of selecting subsequent invasive genetic tests has significantly declined since NIPT was introduced. However, the effects of NIPT on the detection rates of trisomies 21, 18, and 13 have yet been reported.^[7,8]^

Tangshan Maternal and Children Health Hospital is a primary clinical center certified by the Ministry of Health in Hebei Province, China. This clinical center specializes in screening and diagnosing prenatal chromosomal aneuploidies. NIPT has been recommended as a prenatal aneuploidy screening option for high-risk patients since February 2013 in this hospital. We aimed to understand the effect of this additional option on women's preferences regarding invasive diagnostic testing and prenatal chromosomal aneuploidy detection during pregnancy. We performed a retrospective cohort study to evaluate these differences for 1 year before and after NIPT was introduced to clinical practice. We summarized the effects of NIPT and provided insights into the clinical availability and limitation of this approach. We also aimed to determine the mechanism by which clinicians implement such tests in the absence of prenatal screening policies.

## 2 Methods

### 2.1 Participants

The study was approved by the Ethics Committee of Tangshan Maternal and Children Health Hospital and all participants were given informed written consent when they decide to accept or decline whatever test.

This study included all women with a high risk of fetal aneuploidy between 11 and 30 weeks of gestational age visited our hospital for counseling and tests from January 2012 to February 2014. An increased risk of fetal aneuploidy included the following conditions: advanced maternal age (advanced maternal age [AMA]; ≥35 years) at the expected time of delivery, abnormal maternal serum screening (aMSS), family history of abnormal pregnancies including children with Down syndrome and intrauterine fetal death (FH), abnormal ultrasound findings (aUS) suggestive of increased aneuploidy risk, and one parent as a carrier of a chromosomal abnormality. For maternal serum screening, an increased risk of fetal aneuploidy included patients with a risk of >1 in 300 (high risk) and those with risks from 1 in 300 to 1 in 1000 (intermediate risk). US findings considered suggestive of aneuploidy included first-trimester (11–14 weeks) nuchal translucency (NT) ≥3.0 mm or cystic hygroma colli. Second trimester (18–24 weeks) features included nuchal skin-fold thickness ≥6 mm, choroid plexus cysts, absent nasal bone, short humerus less than the fifth percentile for gestational age, short femur less than the fifth percentile for gestational age, echogenic intracardiac focus, echogenic bowel, double-bubble sign, congenital heart defect, or other major congenital anomaly. If two or more indications were detected, the one suggesting a greater risk of chromosomal aberration was chosen. Patients with multiple gestations and pregnancies via in vitro fertilization were excluded.

### 2.2 Pretest counseling

The patients were provided with pretests careful and detailed counseling by a board-certified genetic counselor regarding the increased risk associated with chromosomal aneuploidies on the basis of their maternal age, prior prenatal screening test results, and pregnancy histories. Written informed consents were obtained before and after they underwent further prenatal tests.

### 2.3 Prenatal screening-diagnosis workflow

The patients were recommended invasive prenatal diagnosis with chorionic villus sampling, amniocentesis, cordocentesis, or option to decline further testing between January 2012 and January 2013. In early February 2013, these patients were also provided the option of NIPT. The consent for the prenatal test and other procedures was based on the voluntary decision of patients and was free of costs. The patients were advised of the possible risk of aneuploidy during screening and diagnosis. For each pregnancy without karyotyping results, a postnatal follow-up, including details on pregnancy outcomes obtained from pediatric physical assessment records of neonates, was required. Prenatal screening and diagnosis remain noncompulsory and lack government and insurance coverage because of the policies of the healthcare systems in Hebei Province. Thus, patients were required to pay for conventional invasive tests or NIPT.

### 2.4 Report delivery and posttest counseling

NIPT sequencing analysis was conducted immediately for each DNA sample with a turnover time of 7 working days from receipt of the sample at Berry Genomics Co., Ltd. (Beijing, China). Women with positive NIPT results were provided the option of invasive testing covered by insurance. Invasive testing was performed in our hospital with a turnover time of 21 working days from receipt of the sample. Posttest counseling was given to all participants by a board-certified genetic counselor who advised each individual on the basis of their test results.

### 2.5 Statistical analysis

Continuous variables were expressed as mean ± standard deviation. Statistical analyses were conducted in SPSS 17.0 (SPSS Inc., Chicago, IL). Independent *t* test and analysis of variance were performed to evaluate significant differences. A *P* value of <0.05 was considered statistically significant.

## 3 Results

A total of 4255 high-risk women between 11 and 30 weeks of gestational age visited our hospital for counseling and tests from February 2013 to February 2014. Of these women, 36 with multiple gestations and 14 pregnancies via in vitro fertilization were excluded, and 4205 patients were included for analysis. A total of 3056 patients who exhibited eligibility and applied for NIPT from January 2012 to January 2013 were included in a comparison group. Of these patients, 20 with multiple gestations and 18 pregnancies via in vitro fertilization were excluded, and 3018 patients were considered for analysis (Table 1). Women in the two periods were similar in terms of maternal age, gestational age, and risk factors for counseling.

**Table 1:**
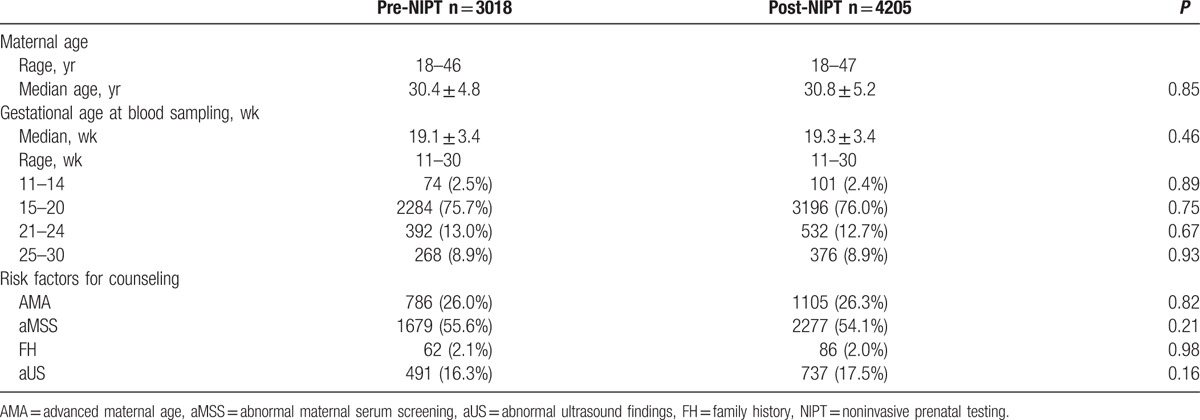
Characteristics of patients referred for prenatal counseling.

In 2013 to 2014, 727 patients (17.3%) underwent invasive testing, 2828 selected NIPT (67.3%), and 650 declined to undergo further testing (15.5%) after NIPT was introduced. A total of 34 cases of trisomies 21, 18, and 13 (0.8%) were found. Of these cases, 24 were found from NIPT and 10 were found from invasive testing. The detection rates of prenatal chromosomal aneuploidies for trisomies 21, 18, and 13 through invasive testing was 13.8%. In 2012 to 2013, 565 patients (18.7%) underwent invasive testing and 2453 declined to undergo further testing (81.3%). A total of 7 cases of trisomies 21, 18, and 13 were detected (0.2%). The detection rates of prenatal chromosomal aneuploidies for trisomies 21, 18, and 13 through invasive testing was 12.4%. After NIPT was introduced, the number of participants who declined to undergo further testing significantly decreased (81.3% vs. 15.5%, *P* < 0.001). Moreover, the detection rates of trisomies 21, 18, and 13 through NIPT significantly increased (0.2% vs. 0.8%, *P* < 0.001). By comparison, the overall rate of invasive testing remained unchanged (18.7% vs. 17.3%, *P* = 0.12) and the detection rates of prenatal chromosomal aneuploidies for trisomies 21, 18, and 13 through invasive testing remained unchanged (13.8% vs. 12.4%, *P* = 0.83) (Table 2).

**Table 2:**
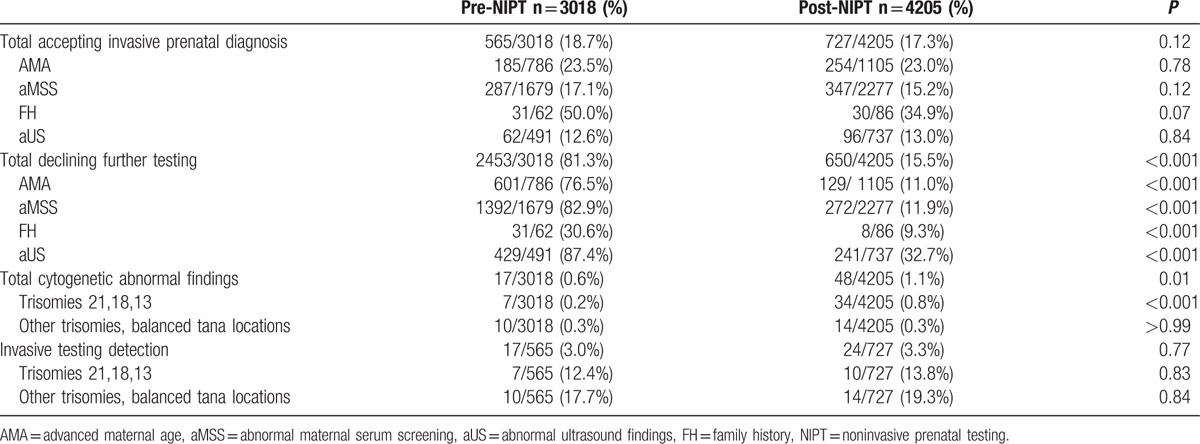
Rates of accepting or declining invasive prenatal diagnosis or cytogenetic abnormal findings.

Of the 2828 patients who selected NIPT, 4 failed subsequently because of a low fetal DNA fraction. Of the 2824 remaining patients who yielded a risk score, 2102 were <35 years old (74.4%) and 722 were ≥35 years old (25.6%). The mean (range) age of the pregnant women was 30.5 years (18–47), the mean (range) gestational age at the time of testing was 19.2 weeks (11–30), and the mean (range) body weight was 63.1 kg (37–99). Indications for testing were AMA, aMSS, FH, and aUS in 25.6% (722/2824), 58.8% (1662/2824), 1.4% (40/2824), and 14.2% (400/2824) of pregnancies, respectively (Table 3).

**Table 3:**
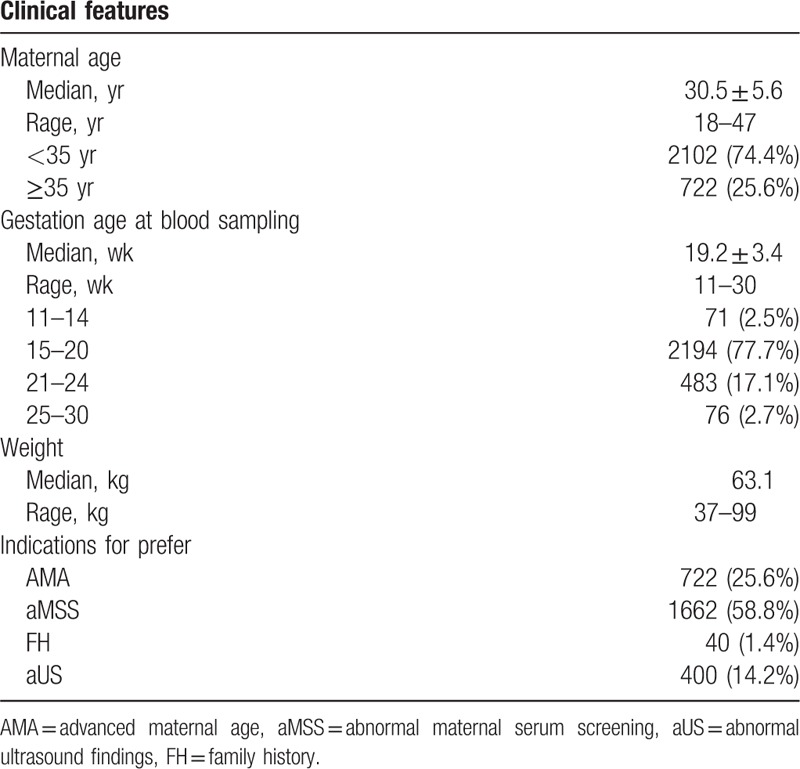
Characteristics of 2824 women who accepted noninvasive prenatal testing.

A total of 31 NIPT cases tested positive, with a positive test rate of 1.1% (31/2824); of these cases, 21 tested positive for trisomy 21, 8 tested positive for trisomy 18, and 2 tested positive for trisomy 13. Of these positive cases, 26 (83.9%) selected further invasive procedure, 2 (6.5%) resulted in intrauterine fetal death (IUFD), and 3 (9.7%) cases refused to undergo subsequence invasive procedures. Of the 21 women positive for trisomy 21, 18 opted to undergo the subsequent invasive procedure, and they were validated by sequence karyotyping; 1 with aMSS resulted in IUFD after the NIPT test result was reported; 1 with AMA and 1 with aUS refused to undergo the subsequent invasive procedure and opted to terminate the pregnancy. Of the 8 women positive for trisomy 18, 6 underwent further invasive procedures, and the results showed that 5 cases manifested trisomy 18 and 1 case exhibited euploid karyotypes (false positive NIPT result); 1 case with AMA resulted in IUFD before the NIPT test results were reported; and 1 case with aMSS refused to undergo further invasive procedures and opted to terminate the pregnancy. Furthermore, 2 patients with positive NIPT for trisomy 13 underwent the subsequent invasive procedures and 1 patient tested positive for trisomy 13. The other patient yielded euploid karyotypes (false positive NIPT result) (Table 4).

**Table 4:**
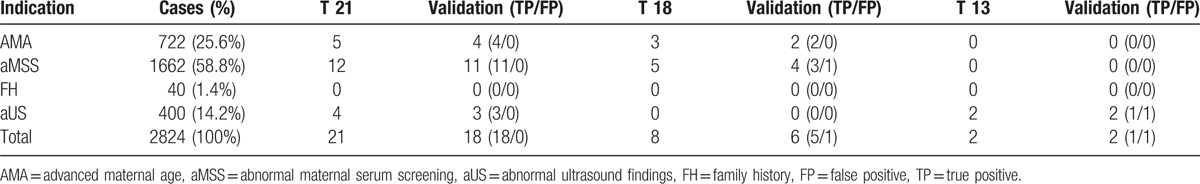
Positive test outcomes of noninvasive prenatal testing and karyotyping validation for 2824 pregnancies.

Outcome data were available for 2762 women within the study for each pregnancy accepted into the NIPT test. No women gave birth to children with trisomies 21, 18, or 13. A total of 2788 pregnancy outcomes (2762 follow-ups and 26 cases with karyotyping results) were available for analysis. A total of 18 cases of trisomy 21, 5 cases of trisomy 18, and 1 case of trisomy 13 were detected; and 1 false positive case for trisomy 18 and 1 trisomy 13 were also found. The sensitivity for the 3 trisomies was 100%. The clinical specificity for trisomy 21 was also 100%. For trisomies 18 and 13, the specificities were 99.9%. For trisomies 21, 18, and 13, the positive predictive values of NIPT results were 100%, 83.3%, and 50.0%, respectively, and the false positive rates were 0% and 0.04% (Table 5).

**Table 5:**
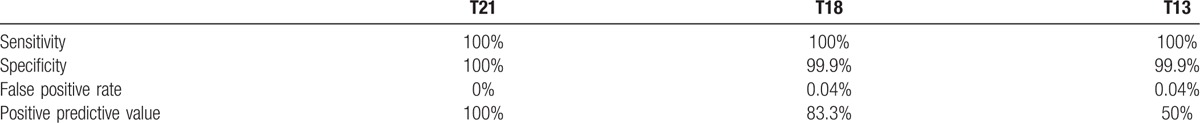
Overall detection rates for trisomies 21, 18, and 13 through noninvasive prenatal testing.

## 4 Discussion

We observed a significant decrease in the number of participants who declined to undergo further testing in high-risk pregnancies after NIPT was introduced. The NIPT introduction has improved the overall efficiency of prenatal detection for trisomies 21, 18, and 13. Within the study period, the rate of invasive testing before and after the introduction of NIPT remained unchanged. Our results are consistent with those of Manegold-Brauer's team.^[9]^ But are inconsistent with those of other studies that reported a significant reduction in the rate of invasive procedures.^[7,10–13]^ We postulate two reasons for these differences. First, pregnancies with fetal structural anomalies and parents who are carriers of chromosomal abnormalities were not advised to select NIPT in our hospital and were recommended with invasive testing because of a high risk of common trisomies, such as 21, 18, and 13, and other less frequent aneuploidies.^[14–16]^ Second, these findings may be attributed to different health-care systems and socioeconomic factors.^[17]^

In our setting, the preference for NIPT by 67.3% (2828/4205) of the women included in our study was much higher than that in other studies, which documented 20% to 30% of women who opted for NIPT.^[10–13]^ Before NIPT was implemented, fetal chromosomal screening in our hospital was highly dependented on invasive prenatal diagnosis testing. Historically, the invasive diagnostic testing rates in our hospital have been lower than those in hospitals in other countries.^[7,11]^ Limited medical resources, such as clinic space, instruments, and certificated physicians available for invasive procedures in our hospital, fail to match the increased demand of patients for prenatal diagnosis tests. In Chinese prenatal diagnosis centers, patients experience difficulty in scheduling invasive procedures within their appropriate gestational age because of medical resource shortage.^[18,19]^ With NIPT in clinical settings, many pregnant women can participate in the detection of prenatal chromosomal aberrations.^[18]^ The rate of women who declined to undergo follow-up testing was significantly decreased after NIPT was introduced (81.3% versus 15.5%, *P* < 0.001), and this finding is consistent with those of Chetty et al.^[7]^ Moreover, a significant increase in the detection rate of trisomies 21, 18, and 13 was found after NIPT became available in clinical settings (0.2% vs. 0.8%, *P* < 0.001). Our experience confirmed that the clinical practice of NIPT provides an efficient workflow in the prenatal diagnosis of chromosomal aneuploidies.

In our study population, NIPT exhibited high sensitivity and specificity (100% and 99.9%). However, two women had fetuses with normal karyotypes among those who were NIPT positive in this study (false positive). This finding indicated the importance of confirming the positive NIPT result by undergoing follow-up invasive procedures. Mennuti et al^[20]^ and Wang et al^[21]^ published cases with inconsistent results between NIPT and cytogenetic testing of pregnancy. These results confirmed that the use of NIPT is more effective as a screening tool than as a diagnostic tool.^[8]^ Although we strongly recommend patients to confirm all abnormal NIPT, 3 (9.7%) of the 31 women with positive NIPT refused to undergo further invasive confirmatory procedures and opted to terminate their pregnancies. In our hospital, all patients were provided pretest careful, detailed counseling, and written informed consent before and after they stated their preferences for further prenatal testing. Without adequate genetic counseling, many patients likely decline to undergo further tests. Therefore, NIPT should be rationally integrated into current practice and in conjunction with appropriate counseling.^[22]^

The potential effectiveness of NIPT is good. However, the mechanism by which this test can be integrated into current clinical practice should be determined. If NIPT remains more expensive than conventional maternal serum screening, expenses possibly impede the introduction of NIPT as the first test in public.^[23]^ Gil et al^[24]^ suggested that NIPT can be recommended as an alternative screening after the first trimester screening (FTS) results are obtained. In our hospital, aMSS is the main basis for counseling and testing referrals before and after NIPT is introduced. Among the 2824 patients who underwent NIPT, 1244 exhibited a high risk and 418 manifested an intermediate risk. The detection rates of prenatal chromosomal aneuploidies for trisomies 21, 18, and 13 were 0.9% (11/1244) and 0.7% (3/418), but the two groups did not significantly differ (0.9% vs. 0.7%, *P* = 0.75). Currently, various guidelines, including the December 2012 Guideline of the American College of Obstetricians and Gynecologists, state that NIPT should not be offered to low-risk women.^[25]^ However, these guidelines may be modified because data showing very high detection rates for Down syndrome from low-risk women have accumulated.^[26–28]^ Tan^[29]^ recommended that combined FTS should be recommended for all pregnant women and alternative tests with NIPT should be offered for those screened to yield a risk of 1 in 101 to 1 in 1000 at a population level; this initiative is cost-effective and suitable for current clinical settings.

With NIPT, ultrasonography remains an important adjunct to prenatal screening.^[30]^ NT and other soft markers found in the second-trimester ultrasound can be used as genetic screening tools.^[31,32]^ Ultrasound soft markers are significantly associated with chromosomal aneuploidies. In addition to costs, unnecessary anxiety may be avoided when women prefer NIPT.^[33]^ In a screening model incorporating NIPT, which yields a substantially lower miscarriage risk, the benefits of adjusting the risk cut-off should be reassessed. Reddy et al^[31]^ recommended that soft markers may be irrelevant in the absence of an elevated a priori risk for fetal aneuploidy, especially after a normal NIPT is introduced.

Noninvasive prenatal testing can be recommended as early as 10 weeks of gestational age.^[34]^ We offered this test at gestational ages between 11 and 30 weeks in our hospital mainly because second maternal serum screening combined with ultrasound examination is the most widely adopted screening protocol in mainland China. We discourage patients with a gestational age of over 30 weeks to select NIPT because of possible positive outcomes and subsequent invasive test confirmation time cost.

This study is characterized by potential limitations that must be considered. First, we employed a retrospective study design, which limits our ability to determine the precise factor influencing a given testing decision. Second, various factors, including individual women's clinical conditions, economic consideration, and prior NIPT knowledge, may affect patients’ decisions, and these factors should be evaluated in future studies. We will extend our study for a longer period to confirm the consistency of results.

In conclusion, our results indicated that NIPT significantly affects the field of prenatal aneuploidy testing. The number of pregnant women who participate in the detection of prenatal chromosomal aberrations has increased and detection efficiencies have improved through NIPT combined with conventional prenatal screening and diagnosis tests. Our study provided practical information for hospitals to integrate and apply NIPT into current prenatal screening-diagnosis workflow in developing countries.

.
